# Infarct quantification using 3D inversion recovery and 2D phase sensitive inversion recovery, validation in patients and ex vivo

**DOI:** 10.1186/1532-429X-14-S1-P37

**Published:** 2012-02-01

**Authors:** Robert Jablonowski, David Nordlund, Henrik Engblom, Joey F Ubachs, Mikael Kanski, Hakan Arheden, Marcus Carlsson

**Affiliations:** 1 Clinical Physiology, Lund University, Skåne University Hospital, Lund, Sweden

## Summary

Infarct quantification with 3D- and 2D-LGE gives similar results in vivo with a very low bias. IR LGE-sequences optimized for in vivo use yield an overestimation of infarct size ex vivo.

## Background

Cardiac-MR (CMR) is the gold standard for quantifying myocardial infarction using late gadolinium enhancement (LGE) technique. Both 2D- and 3D-LGE-sequences are used in clinical practise and in clinical and experimental studies for infarct quantification. Therefore the aim of this study was to investigate if image acquisition with 2D- and 3D-LGE show the same infarct size in patients and ex vivo.

## Methods

26 patients with previous myocardial infarction who underwent a CMR scan were included. Images were acquired 10-20 minutes after an injection of 0.2 mmol/kg Gadolinium-based contrast agent. Two LGE-sequences, 3D-inversion recovery (IR) and 2D-phase-sensitive (PS) IR, were used in all patients to quantify infarction size. Three patients were excluded because of poor image quality due to breathing artefacts and inadequate nulling of the myocardium. Furthermore, six pigs with reperfused infarction in the left anterior descending artery (40 minutes occlusion and 4 hours of reperfusion) were scanned with 3D-LGE and 2D-PSIR ex vivo. A high resolution T1-sequence was used as reference for the infarct quantification ex vivo. The image analysis was done using Segment (http://segment.heiberg.se). Pearson correlation analysis and bias according to Bland-Altman was used for comparison of infarct size with different LGE-sequences.

## Results

**Figure 1:**
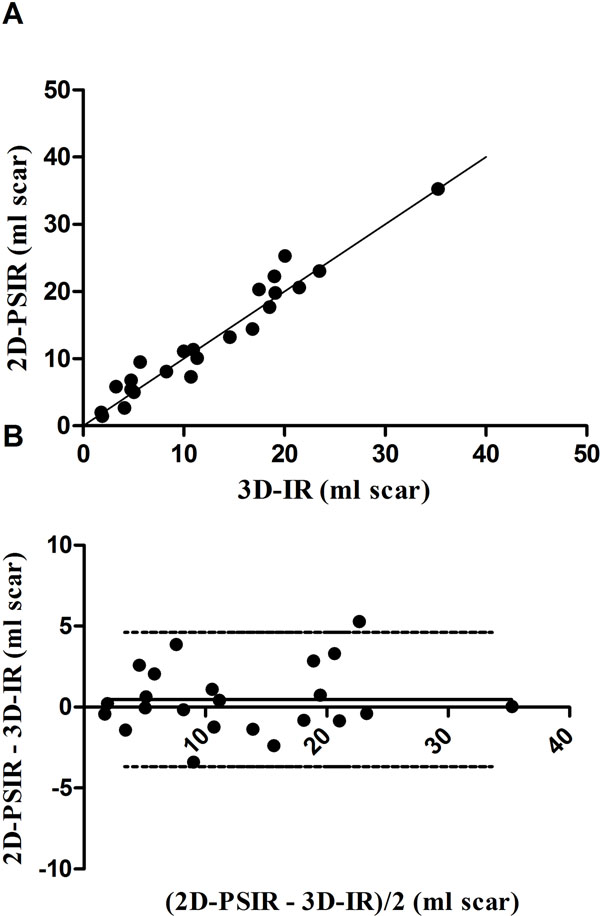
Agreement between 2D-PSIR and 3D-IR. (A) 2D-PSIR versus 3D-IR (r^2^ = 0.94) and the line of identity. (B) The limits of agreement between the two LGE-techniques. The difference between the two methods was 0.47 ± 2.1 ml scar. Solid line = mean difference; dashed lines = ± 2 SD.

Infarct size in vivo using 3D- and 2D-LGE showed high correlation and low bias for both LGE-sequences both in absolute volume of infarct (r^2^ = 0.94, bias 0.47 ± 2.1 ml, Figure 1) and infarct size as part of the left ventricular mass (LVM) (r^2^ = 0.90, bias 0.16 ± 2.0%). Interobserver variability for infarct volume was 0.95 ± 2.9 ml for 3D-LGE and -0.78 ± 2.8 ml for 2D-LGE. The 3D- and 2D-LGE-sequences ex vivo correlated well (r^2^ = 0.81, bias 0.025 ± 2.7 %) for infarct size as part of the LVM. The IR LGE-sequences overestimated infarct size as part of the LVM ex vivo compared to the high resolution T1-sequence ( bias 5.9 ± 2.1 %, 6.4 ± 2.2 % for 2D-PSIR and 3D-IR respectively, p<0.05 for both).

## Conclusions

Infarct quantification with 3D- and 2D-LGE gives similar results in vivo with a very low bias. IR LGE-sequences optimized for in vivo use yield an overestimation of infarct size ex vivo.

## Funding

None.

